# TRPA1 Mediates Mechanical Sensitization in Nociceptors during Inflammation

**DOI:** 10.1371/journal.pone.0043597

**Published:** 2012-08-23

**Authors:** Richard C. Lennertz, Elena A. Kossyreva, Amanda K. Smith, Cheryl L. Stucky

**Affiliations:** Department of Cell Biology, Neurobiology and Anatomy, Medical College of Wisconsin, Milwaukee, Wisconsin, United States of America; University of Cincinnatti, United States of America

## Abstract

Inflammation is a part of the body’s natural response to tissue injury which initiates the healing process. Unfortunately, inflammation is frequently painful and leads to hypersensitivity to mechanical stimuli, which is difficult to treat clinically. While it is well established that altered sensory processing in the spinal cord contributes to mechanical hypersensitivity (central sensitization), it is still debated whether primary afferent neurons become sensitized to mechanical stimuli after tissue inflammation. We induced inflammation in C57BL/6 mice via intraplantar injection of Complete Freund’s Adjuvant. Cutaneous C fibers exhibited increased action potential firing to suprathreshold mechanical stimuli. We found that abnormal responses to intense mechanical stimuli were completely suppressed by acute incubation of the receptive terminals with the TRPA1 inhibitor, HC-030031. Further, elevated responses were predominantly exhibited by a specific subgroup of C fibers, which we determined to be C-Mechano Cold sensitive fibers. Thus, in the presence of HC-030031, C fiber mechanical responses in inflamed mice were not different than responses in saline-injected controls. We also demonstrate that injection of the HC-030031 compound into the hind paw of inflamed mice alleviates behavioral mechanical hyperalgesia without affecting heat hyperalgesia. Further, we pharmacologically anesthetized the TRPA1-expressing fibers *in vivo* by co-injecting the membrane-impermeable sodium channel inhibitor QX-314 and the TRPA1 agonist cinnamaldehyde into the hind paw. This approach also alleviated behavioral mechanical hyperalgesia in inflamed mice but left heat hypersensitivity intact. Our findings indicate that C-Mechano Cold sensitive fibers exhibit enhanced firing to suprathreshold mechanical stimuli in a TRPA1-dependent manner during inflammation, and that input from these fibers drives mechanical hyperalgesia in inflamed mice.

## Introduction

Inflammation is a complex immune response that occurs in response to tissue injury and initiates tissue healing. This process is orchestrated by a cascade of events involving immune cells and inflammatory mediators, which also initiates a side effect of inflammation, inflammatory pain, by sensitizing and activating sensory nerve fibers. Although inflammatory pain reinforces behaviors that avoid further tissue injury, it can become severe, unduly restrict daily activities and reduce the quality of life. Hypersensitivity to mechanical stimuli such as gentle touch or normal limb movement is one of the most troublesome aspects of inflammatory pain and lacks effective clinical treatments.

Understanding the molecular machinery that underlies detection of tactile stimuli is a major frontier in somatosensory research. The Transient Receptor Potential Ankyrin 1 (TRPA1) is one current candidate for participation in mechanosensation. The unique 14–18 ankyrin repeats at the N terminus of TRPA1 initially led to a hypothesis that this region may function as a molecular spring that tethers the channel to the cytoskeletal matrix, thereby serving as a mechanical gate [1]. Several levels of evidence indicate that TRPA1 contributes to normal somatosensory responses to mechanical stimuli in mammals. On a cellular level, TRPA1 has been shown to contribute to mechanically-gated currents in isolated sensory neurons [2], [3]. At sensory terminals in the skin, either genetic ablation or pharmacologic inhibition of TRPA1 reduces mechanical firing in nociceptive sensory afferents [4], [5]. In the CNS, TRPA1 receptors facilitate the transmission of intense peripheral mechanical stimuli to the spinal cord [6]. Further, at a behavioral level, genetic ablation of TRPA1 results in decreased sensitivity to intense mechanical force [7].

Growing evidence suggests that TRPA1 plays an integral role in the both the development and maintenance of inflammatory mechanical hyperalgesia. First, the development of mechanical hyperalgesia coincides with an upregulation of TRPA1 expression in the spinal cord and dorsal root ganglia [8]. Indeed, TRPA1 antagonists prevent both the increase in TRPA1 expression and the enhanced firing of spinal neurons to mechanical stimuli after inflammation [6], [9]. Behaviorally, TRPA1 antagonists prevent the development of and reverse established mechanical hyperalgesia after inflammation [9]–[11]. A number of endogenous inflammatory mediators also activate or sensitize TRPA1. For example, 4-hydroxy-2-nonenal and 15-delta prostaglandin J_2_ can directly activate TRPA1 [12]–[14], whereas bradykinin, serotonin and ATP can act on G protein receptors that indirectly activate TRPA1 [15], [16]. Further, 5,6-epoxyeicosatrienoic acid (EET) has been shown to influence sensory neuron excitation in response to some noxious stimuli by activating TRPA1 [17].

While evidence indicates that TRPA1 can contribute to inflammatory mechanical hyperalgesia by facilitating the transmission of mechanical stimuli to the spinal cord [6], it is not known whether TRPA1 modulates the response properties of the peripheral terminals of sensory neurons during inflammation. Furthermore, while central sensitization is known to contribute to mechanical hyperalgesia, it is not clear whether primary afferent terminals are also sensitized to mechanical stimuli after inflammation and thereby contribute to driving the mechanical behavioral hypersensitivity. There is much controversy in this regard, as some studies demonstrate mechanical sensitization of primary afferents after inflammation [18], [19], while others do not [20], [21].

Here, we investigated the contribution of TRPA1 on cutaneous sensory terminals to inflammatory mechanical hyperalgesia. We show that Complete Freund’s Adjuvant (CFA)-induced inflammation markedly increased responses to mechanical stimuli in a specific subset of C fibers that are sensitive to both mechanical and cold stimuli. Acute pharmacological inhibition of TRPA1 at the receptive terminal normalized C fiber responses to mechanical stimuli. Further, either the anesthetization of TRPA1-expressing nerve fibers or the pharmacologic inhibition of TRPA1 in the hind paw alleviated behavioral mechanical, but not heat, hypersensitivity in inflamed mice. These data strongly support a role for TRPA1 in sensitizing C fibers to intense force, and for these afferent fibers in driving behavioral mechanical hyperalgesia during inflammation.

## Results

### Inflammation Sensitizes C Fibers to Suprathreshold Mechanical Stimuli

**Figure 1:**
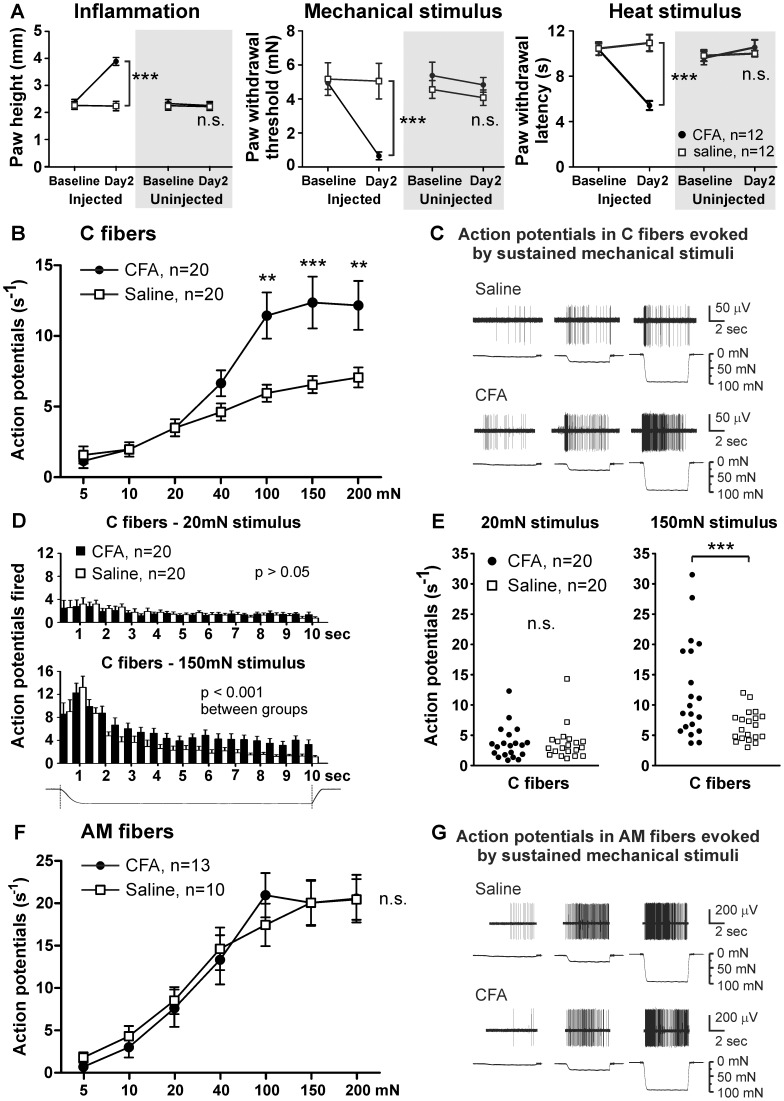
CFA-induced inflammation increases suprathreshold mechanical responses in C fibers. *A*, Comparisons of average paw height (*left*), paw withdrawal threshold for mechanical stimuli (*center*) and paw withdrawal latency from a noxious heat stimulus (*right*) in both the injected and uninjected hind paws of saline-injected and CFA-injected mice. *B*, Force-response curve for C fibers from CFA-injected and saline-injected mice. *C*, Example C fiber response from a saline-injected mouse (*top*) and CFA-injected mouse (*bottom*) to 5, 20 and 100 mN mechanical stimuli. A second fiber with a low-amplitude waveform is noticeable in the bottom trace. *D*, Action potentials fired during a 20 mN (*top*) and 150 mN (*bottom*) mechanical stimulus, displayed in 0.5 sec bins. *E*, Scatterplot of individual C fiber responses to a 20 mN (*left*) and 150 mN (*right*) mechanical stimulus. *F*, Force-response curve for AM fibers from CFA-injected and saline-injected mice. *G*, Example AM fiber response from a saline-injected mouse (*top*) and CFA-injected mouse (*bottom*) to 5, 20 and 100 mN mechanical stimuli. *, two-way ANOVA followed by a Bonferroni post-hoc test.

CFA injection into the mouse hind paw induced marked inflammation, resulting in subsequent mechanical and heat hypersensitivity that peaked at 2 days post injection (Fig. 1A) [26]. To determine whether inflammation sensitizes primary afferent fibers to mechanical stimuli, we performed teased fiber recordings in skin-nerve preparations taken from mice 48–72 hrs after injection of CFA or saline. We performed these recordings from the sural nerve because the innervation territory of this nerve extends to the lateral plantar surface of the hind paw and overlaps with the skin region that is tested by our behavioral assays. Thus, we could directly compare our results from electrophysiological recordings to the data obtained from behavioral assays.

C fibers from inflamed mice fired significantly more action potentials in response to suprathreshold stimuli (100–200 mN) compared to C fibers from saline-injected mice (p<0.001, Fig. 1B, 1C). In fact, C fibers from inflamed mice fired almost twice as many action potentials at forces above 100 mN (Fig. 1B). By examining the adaptation of the mechanical response throughout the stimulus, we found that C fibers from inflamed mice appear to maintain higher rates of firing during the static phase of the stimulus (1.5 through 10 sec; p<0.001, Fig. 1D). However, C fibers from inflamed and control mice reached similar maximum rates of firing during the initial phase of the stimulus (Fig. 1D). Interestingly, some C fibers exhibited exceptionally large responses to intense mechanical stimuli (150 mN, 14–32 Action Potentials APs/s), whereas other C fibers exhibited similar responses compared to saline controls (150 mN, <12 APs/s; p<0.05, Fig. 1E).

The mechanical thresholds of C fibers from inflamed mice (median 4; range: 4–6.8 mN) were not different than those from saline controls (median 4; range: 4–6.8 mN; p>0.05). This is consistent with the finding that action potential firing is not different between these groups at low-intensity sustained forces (Fig. 1B).

Unlike C fibers, AM fibers did not exhibit sensitization to suprathreshold mechanical stimuli (Fig. 1F, 1G). Also, there was no difference in mechanical thresholds in AM fibers from inflamed mice (CFA: median 6.8; range: 5.4–6.8 mN vs. saline: median 6.8; range: 4.0–6.8 mN; p>0.05). These data demonstrate that inflammation markedly sensitizes C fibers to suprathreshold mechanical stimuli without altering their mechanical thresholds, and that Aδ nociceptors are not sensitized by inflammation.

### TRPA1 Channel Inhibition Completely Reverses Mechanical Sensitization in C Fibers

Next we investigated mechanisms that underlie mechanical sensitization. The TRPA1 channel has been implicated in mediating mechanical hypersensitivity after tissue injury. The TRPA1 antagonists AP-18 and HC-030031 have been shown to reduce CFA-induced mechanical hypersensitivity in behavioral assays [9]–[11]. However, the contribution of TRPA1 at the sensory terminals in skin is not known. Therefore, we performed behavioral assays and teased nerve fiber recordings in the presence of HC-030031.

**Figure 2:**
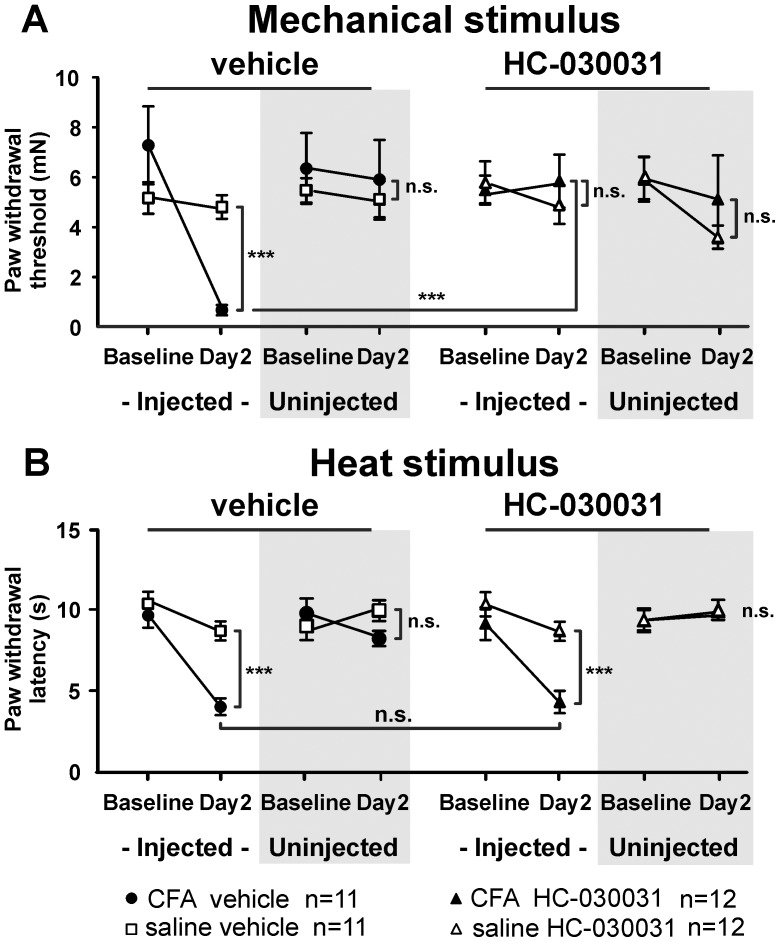
A TRPA1 antagonist, HC-030031, alleviates mechanical hyperalgesia. Treatment groups were divided into CFA+vehicle, saline+vehicle, CFA+HC-030031 and saline+HC-030031 (100 µg HC-030031 in vehicle). *A,* Mechanical paw withdrawal threshold for each group in the injected paw (*white*) and uninjected paw (*grey*). *B,* Thermal paw withdrawal latency for each group in the injected paw (*white*) and uninjected paw (*grey*). *, two-way ANOVA followed by a Bonferroni post-hoc test of all four groups.

Two days after the development of inflammation, HC-030031 (100 µg) was injected into the plantar surface of the hind paw. Local injection of HC-030031 significantly reversed the hypersensitivity caused by CFA in inflamed mice, as seen by the return of paw withdrawal thresholds to baseline levels (p<0.001; Fig. 2A; horizontal bar). Further, inflamed mice treated with HC-030031 no longer exhibited mechanical hypersensitivity when compared to saline-injected controls (p>0.05; Fig. 2A; vertical bar, non-shaded region). To our knowledge, behavioral testing with heat has not been previously reported in the presence of HC-030031. Unlike its reversal of mechanical hypersensitivity, injection of HC-030031 did not attenuate heat hypersensitivity in inflamed mice (p>0.05), and inflamed mice continued to exhibit heat hypersensitivity compared to saline-injected controls (p<0.001; Fig. 2B, horizontal bar). These data suggest that the TRPA1 channel mediates mechanical hypersensitivity, but not heat hypersensitivity, during inflammation.

**Figure 3:**
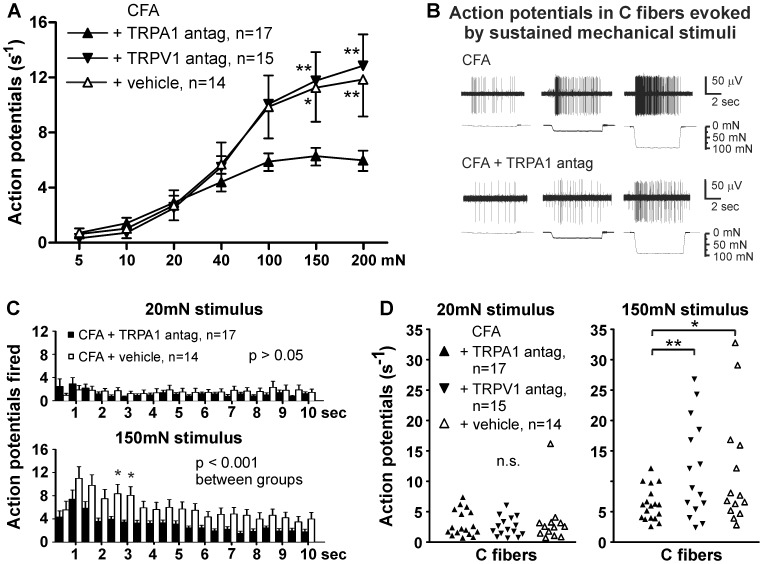
A TRPA1 antagonist, HC-030031, completely normalizes C fiber responses to mechanical stimuli. *A*, Force-response curves for C fibers from CFA-injected mice in the presence of either HC-030031 (30 µM) or vehicle. *B,* Example C fiber response from a CFA-injected mouse (*top*) and a CFA-injected mouse in the presence of HC-030031 (*bottom*) to 5, 20 and 100 mN mechanical stimuli. A smaller, spontaneously-active fiber is noticeable in the bottom trace. *C,* Action potentials fired during a 20 mN (*top*) and 150 mN (*bottom*) mechanical stimulus in the presence in CFA-injected mice, displayed in 0.5 sec bins. *D,* Scatterplot of individual C fiber responses to a 20 mN (*left*) and 150 mN (*right*) mechanical stimulus in CFA-injected mice. *, two-way ANOVA followed by a Bonferroni post-hoc test.

In conjunction with behavioral assays, HC-030031 was used in teased nerve fiber recordings to determine the role of TRPA1 at the peripheral nerve terminals in skin. C fiber receptive fields were acutely treated with HC-030031 or vehicle for 2 min and tested with force in the presence of compound. HC-030031 completely reversed the enhanced mechanical firing in inflamed mice (p<0.001, Fig. 3A, 3B). In fact, mechanical responses in C fibers treated with HC-030031 were no different than responses in C fibers from saline-injected mice (p>0.05; Fig. 3A vs. Fig. 1A). Since TRPA1 is often co-localized and may interact with the Transient Receptor Potential Vanilloid 1 (TRPV1) channel, we also tested mechanical responses in the presence of the TRPV1 antagonist A-425619. However, mechanical responses in inflamed mice were not reduced in the presence of A-425619 (p>0.05, Fig. 3A). Vehicle treatment had no effect on mechanical responses in inflamed mice (p>0.05, Fig. 3A vs Fig. 1A). By examining the adaptation of mechanical firing over the duration of the stimulus, we found that the antagonist HC-030031 significantly reduced firing at multiple time points during the sustained phase of the stimulus (p<0.05; Fig. 3C). Furthermore, out of 17 C fibers tested with force in the presence of HC-030031, none exhibited the exceptionally large responses (>14 APs/s) to an intense mechanical stimulus that were observed in the presence of A-425619 or vehicle in inflamed mice (150 mN; Fig. 3D). Instead, the range of their mechanical firing was similar to saline-injected C fibers (Fig. 3D, right vs. Fig. 1D, right). These data indicate that TRPA1 mediates the enhanced action potential firing evoked by intense force at the receptive terminals of C fibers in inflamed mice.

### TRPA1-expressing C Fibers Mediate Inflammatory Mechanical Hypersensitivity

We next set out to confirm the effect of TRPA1 channel inhibition by HC-030031 by assessing the contribution of TRPA1-expressing C fibers to mechanical hypersensitivity in inflamed mice. Here, we employed the constitutively-charged lidocaine analogue QX-314. Unlike lidocaine, QX-314 does not diffuse across the neuronal plasma membrane, but requires a port of entry into the nerve. Indeed, QX-314 has previously been used *in vivo* together with capsaicin; capsaicin caused opening of TRPV1 channels, allowing QX-314 to enter and anesthetize TRPV1-expressing afferents [27]. Like TRPV1 channels, activated TRPA1 channels undergo pore dilation large enough to allow entry of molecules such as QX-314 [28]. Therefore, we co-injected QX-314 and the TRPA1 agonist cinnamaldehyde into the hind paw in order to selectively anesthetize TRPA1-expressing nerve fibers in inflamed or non-inflamed mice.

**Figure 4:**
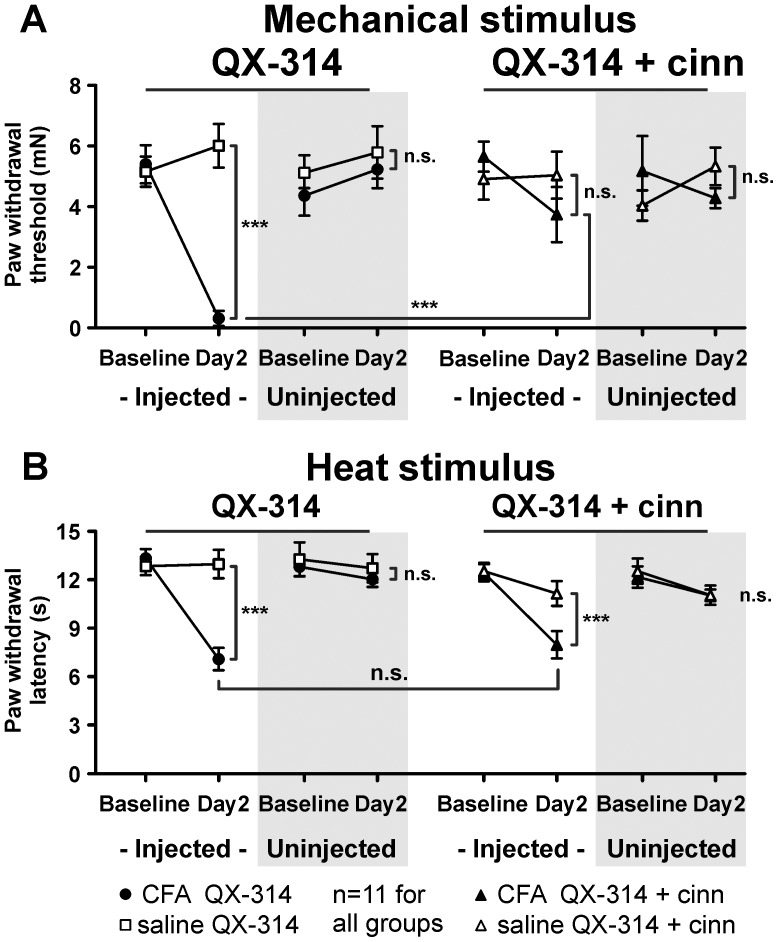
TRPA1-expressing fibers drive behavioral mechanical hyperalgesia. Treatment groups were divided into CFA-QX-314, saline-QX-314, CFA-QX-314+cinnamaldehyde (cinn) and saline-QX-314+cinn (concentrations were 0.2% QX and 30 µM cinn). *A,* Mechanical paw withdrawal threshold for each group in the injected paw (*white*) and uninjected paw (*grey*). *B,* Thermal paw withdrawal latency for each group in the injected paw (*white*) and uninjected paw (*grey*). *, two-way ANOVA followed by a Bonferroni post-hoc test of all four groups.

Local co-injection of QX-314 and cinnamaldehyde significantly elevated paw withdrawal thresholds in CFA-injected mice compared to QX-314 alone (p<0.001, Fig. 4A; horizontal bar). In fact, inflamed mice treated with QX-314 and cinnamaldehyde no longer exhibited mechanical hypersensitivity compared to saline-injected controls (p>0.05, Fig. 4A). Importantly, the injection of QX-314 alone did not alter paw withdrawal thresholds in inflamed mice (p<0.001, Fig. 4A). In contrast to the effect on mechanical hypersensitivity, heat hypersensitivity was not alleviated by co-injection of QX-314 and cinnamaldehyde (p>0.05; Fig. 4B; horizontal bar). Inflamed mice treated with QX-314 and cinnamaldehyde continued to exhibit reduced withdrawal latencies to a noxious heat stimulus compared to saline-injected controls (p<0.001, Fig. 4B). The injection of QX-314 alone did not alter paw withdrawal latencies in inflamed mice (p<0.001, Fig. 4B).

These results support the TRPA1 channel inhibition results above by demonstrating that TRPA1-expressing C fibers mediate mechanical hypersensitivity, but not heat hypersensitivity, in inflamed mice.

### Inflammation Sensitizes Mechano Cold Sensitive C Fibers to Mechanical Stimuli

We noted that some C fibers exhibited unusually large responses to mechanical stimuli in inflamed mice (Fig. 1D) and that treatment with the TRPA1 antagonist HC-030031 suppressed these large responses (Fig. 3D). This suggested that a subset of C fibers may mediate the mechanical hypersensitivity in inflamed mice, and that this population of C fibers expresses TRPA1.

**Figure 5:**
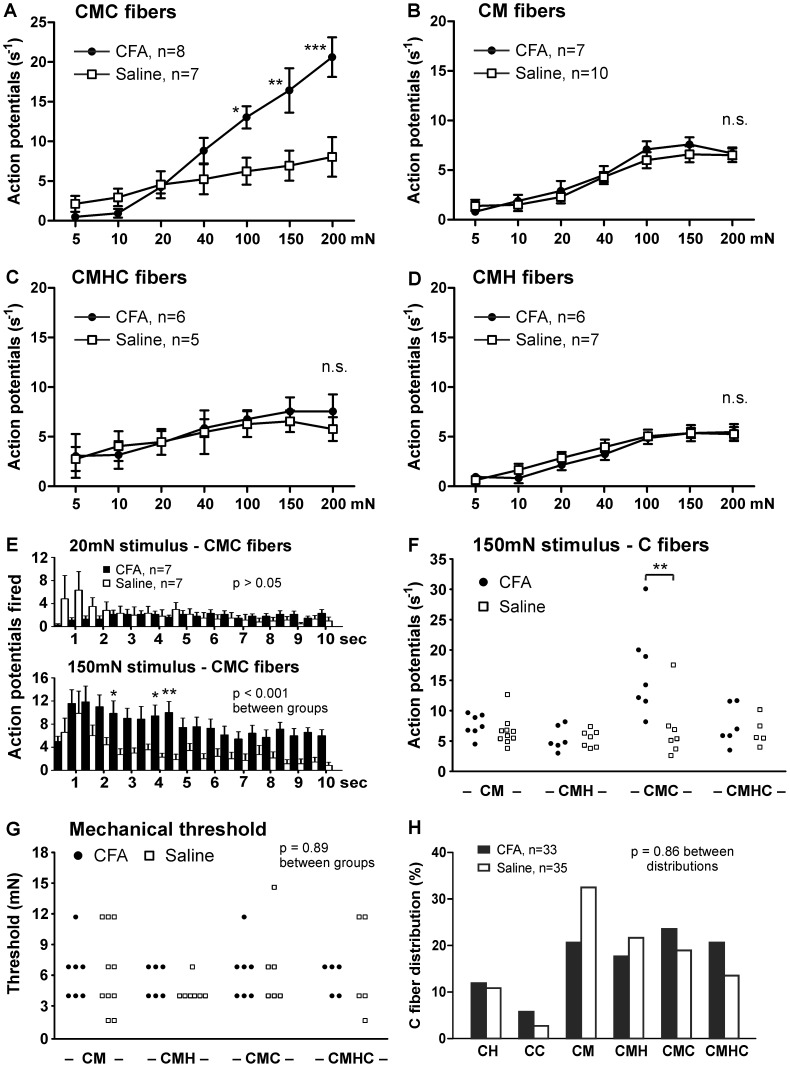
Inflammation increases mechanical responses of CMC fibers. Force-responses curves for *A,* CMC fibers *B*, CM fibers *C*, CMHC fibers and *D*, CMH fibers from CFA-injected and saline-injected mice. *E*, Action potentials fired by CMC fibers during a 20 mN (*top*) and 150 mN (*bottom*) mechanical stimulus, displayed in 0.5 sec bins. *F*, Scatterplot of individual C fiber responses to a 150 mN mechanical stimulus. *G*, Scatterplot of C fiber mechanical threshold, as determined by calibrated von Frey filament. *H*, Distribution of C fiber subtypes characterized from CFA-injected and saline-injected mice. *, two-way ANOVA followed by a Bonferroni post-hoc test.

In order to further assess the population of C fibers that mediates mechanical hypersensitivity in inflamed mice, we performed a separate set of recordings in which C fibers were subtyped according to their sensitivity to mechanical, heat and cold stimuli. We found that C-Mechano Cold (CMC) fibers from inflamed mice showed a striking increase in mechanical firing to suprathreshold force (above 40 mN; Fig. 5A). On the other hand, mechanical responses in C-Mechano only (CM), C-Mechano Heat (CMH) and C-Mechano Heat Cold (CMHC) fibers were similar between inflamed and saline-injected mice (p>0.05, Fig. 5B, 5C, 5D). The CMC fibers responded to 150 mN forces with nearly three times as many action potentials as saline controls (p<0.01, Fig. 5A). While recordings from non-subtyped C fibers suggested a higher sustained rate of firing in inflamed mice (Fig. 1C), this effect was especially pronounced among CMC fibers (p<0.001, Fig. 5E). Additionally, CMC fibers from inflamed mice exhibited the greatest firing to intense force (150 mN) and generally exhibited larger responses than any other subtype of C fiber (p<0.01, Fig. 5F). There was no difference in mechanical thresholds of CM, CMH, CMC or CMHC fibers between CFA-injected and saline-injected mice (p = 0.89, Fig. 5G). Also, there was no shift in the proportion of C fiber subtypes following CFA injection (p = 0.81, Fig. 5H). Together, this suggests that the exceptionally large mechanical responses observed in previous recordings, which were suppressed by the TRPA1 antagonist HC-030031, are responses from CMC fibers.

We did not observe reduced thresholds or increased action potential firing to either cold or heat stimuli in inflamed mice (data not shown). In fact, overall, the action potential responses of C fibers to heat were decreased after inflammation, as shown by Andrew and Greenspan, 1999 [18]. Similarly, the action potential responses to cold were decreased after inflammation. However, we cannot exclude that the absence of thermal sensitization in our study may have been due to desensitization induced by the prior mechanical stimuli.

These data demonstrate that inflammation sensitizes C-Mechano Cold fibers to mechanical stimuli without affecting the mechanical response properties of other cutaneous C fiber subtypes.

## Discussion

The TRPA1 ion channel is expressed on nociceptive sensory neurons where it is sensitive to an array of endogenous pro-inflammatory mediators and contributes to normal afferent mechanical responses, and potentially cold responses [29], [30]. The versatility of this channel has led to considerable interest in TRPA1 as a putative target for novel analgesic agents. Indeed, pharmacological inhibition of TRPA1 reduces the behavioral mechanical hypersensitivity that accompanies inflammation in animal models [9]–[11], and has also been shown to reduce postoperative pain [31]. However, the way in which TRPA1 contributes to sensitization after tissue injury is not known. An accurate assessment of the role of TRPA1 has been complicated by a poor consensus on the physiological underpinnings of mechanical hyperalgesia. In particular, multiple studies have debated whether primary afferent nerves exhibit sensitization to mechanical stimuli after tissue injury. Here, our findings show that a subset of cutaneous primary afferent C fibers exhibits sensitization to mechanical stimuli in inflamed mice. Further, we show that mechanical sensitization is dependent on TRPA1, as either inhibition of TRPA1 channels or anesthetization of TRPA1-expressing nerve fibers alleviated mechanical hypersensitivity in inflamed mice. We believe that our findings clarify the issue of primary afferent nerve sensitization and explain how TRPA1 contributes to inflammatory mechanical hyperalgesia.

### Inflammation Sensitizes C Fibers to Suprathreshold Force

We found that inflammation markedly enhances C fiber responses to intense, suprathreshold force. Although several other studies have reported that C fiber responses to mechanical stimuli do not increase during inflammation [21], [32], [33], the conclusions of these studies were based on mechanical threshold measurements. Indeed, our data indicate that inflammation does not alter C fiber mechanical thresholds but rather amplifies the action potential firing to intense force that is presumably in the noxious range. Furthermore, this increase occurred because C fibers sustained a higher rate of firing throughout the static phase of intense sustained mechanical stimuli.

Our data appear to differ from a recent *in vitro* mouse study by Koerber and colleagues that found no sensitization to suprathreshold force during inflammation [20]. However, the Koerber study examined forces only up to 100 mN whereas our study shows enhanced mechanical responses between 100–200 mN. In addition, we used a 0.8 mm diameter probe whereas the Koerber study used a 1.0 mm diameter probe. Thus, a 100 mN force in the Koerber study delivers the same pressure as a 64 mN force in our study, which falls just below the range at which we observed an increase in mechanical firing. Also, the Koerber study focused on C fibers that were sensitive to heat stimuli, a population that did not exhibit mechanical sensitization in our study. For these reasons, we believe our data are actually consistent with the Koerber et al 2010 study, but go farther in the characterization of primary afferent firing properties after inflammation.

Prior electrophysiology studies use different animal models, techniques and measures to study mechanical sensitization during inflammation. In general, *in vivo* models have found mechanical sensitization in primary afferent fibers, while prior *ex vivo* models have not. This discrepancy has been attributed to factors present *in vivo*, such as edema-induced stretch of the skin, the continuous presence of inflammatory mediators in the milieu of the terminal, and factors released from an intact vasculature. However, we found here that C fibers are indeed sensitized to mechanical stimuli in an *ex vivo* preparation and the enhanced suprathreshold firing can be reversed by acute local treatment with a TRPA1 antagonist. This indicates that mechanical sensitization is an inherent property of the sensory neurons within their cutaneous milieu. Thus, we propose that other differences account for prior discrepancies, particularly differences in the property measured (threshold vs. suprathreshold firing) and the intensity and type of mechanical stimulus used in each study.

### Mechanical Sensitization Occurs in C-Mechano Cold Fibers

A subpopulation of C fibers exhibited dramatically increased responses to intense mechanical stimuli. In fact, some responses were 4–6 times larger than an average response in control mice. We found that these exceptionally large responses were only exhibited by C-Mechano Cold fibers, whereas responses from other subtypes of C fibers were within a normal range. To our knowledge, little has been reported about the role of C-Mechano Cold fibers in normal sensation or inflammatory pain. However, we can draw some conclusions about C-Mechano Cold fibers from our current study. Our teased fiber recordings suggest that C-Mechano Cold fibers express TRPA1, as the TRPA1 antagonist completely suppressed all of the exceptionally large responses to intense mechanical stimuli in C fibers. Our experiments also indicate that C-Mechano Cold fibers are integral to mechanical hyperalgesia because anesthetizing these fibers with the QX-314 anesthetic or inhibiting the TRPA1 channel with an antagonist alleviated mechanical hypersensitivity in inflamed mice. Interestingly, a study of ultraviolet light-induced inflammation also reports elevated suprathreshold mechanical responses in heat-insensitive C fibers, which would include C-Mechano Cold fibers [34]. This suggests that C-Mechano Cold fibers may be involved in other models of tissue injury as well as CFA-induced inflammation. Together, these studies suggest that C-Mechano Cold fibers play an important role in inflammatory mechanical hyperalgesia.

Our study also questions previous reports concerning the distribution of TRPA1 and TRPV1 channels on sensory neurons. Since TRPV1 is a heat-sensitive channel and C-Mechano Cold fibers are not sensitive to heat, this argues that C-Mechano Cold fibers do not express the TRPV1 receptor. This is important in light of other studies that have shown that TRPA1 is extensively co-expressed in approximately 50% of TRPV1-expressing C fibers, but is rarely expressed in C fibers alone [30], [35]–[37]. Recently, however, Malin and colleagues demonstrated that 10% of cutaneous afferents respond to TRPA1 but not TRPV1 agonists, suggesting that there is a population of TRPA1-only cutaneous C fibers in non-injured tissue [38]. Furthermore, since inflammation increases expression of TRPA1 [8], [9], it is possible that TRPA1 may be expressed *de novo* on neurons that do not express TRPV1 after tissue injury. Our data suggest that after inflammation, there is a population of TRPA1-expressing, but not TRPV1-expressing, C fibers that are particularly sensitized to mechanical stimuli.

The distribution of TRPV1 is also important to the conclusions of our study. TRPV1 has been shown to mediate mechanical hypersensitivity after bone cancer, inflammation, nerve injury and sickle cell disease [39]–[42]. However, heat-sensitive C fibers did not exhibit mechanical sensitization in our study. Rather, heat-insensitive fibers, which would not express TRPV1, were sensitized to mechanical stimuli. Further, incubation with a TRPV1 antagonist did not affect mechanical responses in recordings from inflamed mice. In contrast, in a mouse model of sickle cell disease, incubation of cutaneous C fiber terminals with the same TRPV1 antagonist, A-425619, completely reversed the sensitized mechanical firing in sickle C fibers [42]. Therefore, although inflammation is a component of sickle cell disease, the mechanisms driving mechanical sensitization in peripheral inflammation induced via CFA versus sickle cell disease are distinct. Our data here indicate that TRPV1 does not contribute to mechanical sensitization in a CFA model of inflammation.

### Sensory Nerve Fiber Input Plays an Essential Role in Mechanical Hyperalgesia

The TRPA1 antagonist HC-030031 completely alleviated mechanical hypersensitivity in inflamed mice. These results argue that TRPA1 plays an integral role in inflammatory mechanical hyperalgesia and are consistent with previous studies [9]–[11]. Further, we found that the HC-030031 compound has no effect on heat hyperalgesia in inflamed mice, suggesting that while TRPA1 plays an important role in mechanical hyperalgesia, it does not play an essential role in heat hyperalgesia. Instead, as clearly demonstrated by other groups, TRPV1 is a key mediator of inflammatory heat hypersensitivity [43], [44]. Importantly, HC-030031 was injected into the hind paw, targeting TRPA1 channels expressed on the sensory nerve terminals, at a dose that did not ameliorate mechanical hyperalgesia in a previous study [9]. Thus, our results support the conclusion that the increase in mechanical responses exhibited by C fibers is physiologically significant, and that suppressing mechanical hypersensitivity in sensory nerve fibers can alleviate behavioral mechanical hyperalgesia.

We felt that using an alternative approach would further substantiate our findings, as one could question either the specificity of the HC-030031 antagonist or the localization of its effect. Therefore, we targeted TRPA1-expressing fibers using QX-314 and cinnamaldehyde. To our knowledge, this is the first example of QX-314 anesthetization of TRPA1-expressing neurons *in vivo*. This approach was effective using only small quantities of cinnamaldehyde, which increased our confidence that the treatment had a localized effect on cutaneous nerve fibers in the hind paw. Our results also support that input from TRPA1-expressing nerve fibers mediates mechanical hypersensitivity. In addition, our laboratory has previously shown that the HC-030031 compound has no effect on mechanical responses in TRPA1 knockout mice [4]. While these experiments do not preclude the possibility of off-target effects, they support that the TRPA1 channel contributes to mechanical hyperalgesia.

### The Role of TRPA1 in Mechanical Sensation

Our group has previously shown that acute exposure of C fiber receptive fields to the TRPA1 antagonist HC-030031 reduces suprathreshold firing to intense stimuli in non-inflamed mice. Non-inflamed global TRPA1 knockout mice also exhibited reduced mechanical firing in C fibers [4]. In these experiments, TRPA1 influenced mechanical responses during the static phase of the mechanical stimulus, similar to our observations in inflamed mice (Figures 1C, 3C and 5E). First, these experiments suggest that TRPA1 modulates mechanical responses by influencing C fiber adaptation. Second, one may have expected the TRPA1 antagonist to further reduce mechanical responses in inflamed mice below the levels seen in saline-injected mice. The fact that we did not observe a further reduction here in the setting of tissue inflammation may indicate that proteins other than TRPA1 contribute to mechanical sensitization in inflamed mice. Alternatively, this may reflect minor differences in the recording protocol used in each study. Even so, our present study argues that mechanical sensitization does not persist in the acute absence of functional TRPA1. Importantly, both studies agree that TRPA1 is a major regulator of suprathreshold mechanical firing properties.

At this time, there is a limited understanding of the mechanism by which TRPA1 enhances suprathreshold mechanical responses in sensory neurons. TRPA1 can be regulated by cytoplasmic calcium either by direct binding to an EF hand-like motif [45], [46] or indirectly via a calmodulin-independent mechanism [16]. Thus, mechanical stimuli may activate C fibers and result in calcium influx that subsequently activates TRPA1 via intracellular binding. TRPA1 activation, in turn, may result in an enhanced receptor potential. At this time, there is no clear evidence that TRPA1 is directly mechanically sensitive in mammalian cells. However, TRPA1 activation by mechanical stimuli could also result in an enhanced receptor potential. Alternatively, TRPA1 activation by any means may influence action potential generation by enhancing calcium influx and indirectly modulating the function of other membrane proteins [2].

TRPA1 is a promising target for therapeutic intervention in inflammatory pain. In the presence of a TRPA1 antagonist, mechanical responses in C fibers from inflamed mice were no different than mechanical responses in control mice, suggesting that TRPA1 antagonists may be able to inhibit hyperalgesia without interfering with normal mechanical sensation in the setting of tissue inflammation. In fact, inflamed mice exhibited normal mechanical sensitivity when either TRPA1 was pharmacologically inhibited or TRPA1-expressing fibers were anesthetized. In addition to our study which demonstrates the integral role of TRPA1 in primary afferent fibers, other studies suggest an important role for TRPA1 in the spinal cord during inflammation [6], [9]. Thus, an oral TRPA1 antagonist may be effective in reducing inflammatory mechanical hyperalgesia at multiple levels of signal transduction. Along with previous studies, our results highlight the potential of TRPA1 antagonists to reduce mechanical hyperalgesia without abolishing normal tactile acuity and improve the quality of life for people who live with inflammatory pain.

## Methods

### Animals

Adult male C57BL/6 mice (8–12 weeks old), obtained from Jackson Laboratories, were used for all experiments. Mice were housed in group cages, maintained on a 12∶12 hr light-dark cycle, in a controlled environment (21°C) and given unrestricted access to food and water. All procedures were approved by the Institutional Animal Care and Use Committee at the Medical College of Wisconsin and were performed in accordance with the guidelines put forth by the National Institutes of Health (AUA00000383).

### Injections

Mice were briefly anesthetized using inhaled isoflurane. Mice were injected with a 30 µL emulsion of undiluted CFA into the medial left plantar hind paw. The vehicle control group was injected with 30 µL of sterile 0.9% saline solution. Two days after injection, at the peak of hypersensitivity, the magnitude of inflammation was measured at the midpoint of the hind paw using digital calipers (VWR). For one experiment, the membrane-impermeable sodium channel inhibitor lidocaine N-ethyl-bromide, also known as QX-314, (0.2% in saline; 30 µL) was injected with or without the TRPA1 agonist cinnamaldehyde (30 µM) into the left plantar hind paw 2 days post CFA injection. For another experiment, the TRPA1 antagonist HC-030031 (100 µg in 30 µl of 0.5% DMSO and 0.25% Tween-80 in PBS [9]) was injected into the left plantar hind paw 2 days post CFA injection. Vehicle controls were injected with 30 µl 0.5% DMSO and 0.25% Tween-80 in PBS. All behavioral assays were completed between 1 and 4 hours following the QX-314, HC-030031 or vehicle injections. The experimenter was blinded to chemical treatment for all studies.

### Behavioral Assays

For measuring mechanical response thresholds, mice were allowed to acclimate on a wire mesh floor for at least 30 minutes prior to testing. Calibrated von Frey monofilaments (0.22, 0.27, 0.66, 1.63, 4.0, 6.8, 11.7, 14.6 mN; Smith and Nephew, Inc., Germantown, WI) were applied to the medial plantar surface of the hind paw and the 50% paw withdrawal threshold was calculated for each paw using the up-down method [22]. For measuring heat responses, mice were allowed to acclimate on a glass surface for at least 30 min. Radiant noxious heat was applied to the plantar surface of the hind paw and the latency to paw withdrawal was measured for each paw as previously described [23]. For behavioral assays, baseline mechanical threshold and thermal latency measurements were acquired prior to any other experimental procedure. These measurements were then repeated two days following the injection of CFA or vehicle. At least 1 hr was allowed for recovery between conducting mechanical and heat assays. Mice used in behavioral assays were not subsequently used for teased nerve fiber recordings.

### Teased Nerve Fiber Recordings

Between 48 and 72 hours following the injection of CFA or vehicle, mice were anesthetized via inhaled isoflurane and killed. The glabrous and hairy skin from the lateral and plantar surfaces of the hind paw was dissected free along with the innervating sural nerve. The preparation was placed in a tissue bath dermal side up and superfused with synthetic interstitial fluid as previously described [24], [25]. The solution contained the following (mM): 123 NaCl, 3.5 KCl, 0.7 MgSO_4_, 1.7 NaH_2_PO_4_, 2.0 CaCl_2_, 9.5 sodium gluconate, 5.5 glucose, 7.5 sucrose and 10 HEPES; pH 7.45±0.05, 290 mOsm, temperature 32±0.5°C. The solution was continuously aerated with O_2_. Using sharpened forceps, the nerve was desheathed, teased into thin filaments, and placed on a silver extracellular recording electrode. Filaments were split smaller until clean, single unit extracellular recordings could be obtained from isolated receptive fields. The receptive fields were identified using an electrical search stimulus whereby the skin was panned with a needle electrode. Fibers were then characterized according to their conduction velocity as previously described for mouse (Aâ fibers had velocities ≥10 m/s; Aä velocities were between 1.2 and 10 m/s and C fibers had velocities <1.2 m/s) [25]. The mechanical threshold of each fiber was determined using von Frey filaments (0.044 to 147 mN). Baseline activity was recorded for at least 1 minute prior to the application of mechanical or thermal stimuli. Any fiber with ongoing activity ≥0.1 Hz was considered to have spontaneous activity. Fibers were further classified as rapidly adapting (RA) or slowly adapting (SA) based on adaptive properties to force. SA fibers responded throughout a sustained mechanical force, whereas RA fibers responded predominantly at the onset and offset of force. The Aδ fibers were classified as either slowly adapting A-mechanoreceptors (AM) or rapidly adapting Down-hair (D-hair) receptors. The D-hair fibers responded to very low mechanical forces (<1 mN), whereas AM fibers typically exhibited mechanical thresholds ≥4 mN. Furthermore, D-hair receptors had very large receptive fields (up to 8 mm in diameter) whereas AM fibers had very small (≤1 mm) discrete, sensitive receptive field spots. C fibers were all slowly adapting. The minimum action potential amplitude for fibers was 3-fold greater than the noise level.

Next, characterized nerve fibers were activated with mechanical stimuli by using a feedback-controlled computer-driven mechanical stimulator with a ceramic probe 0.8 mm in diameter and with a smooth, flat end. Increasing mechanical forces (5, 10, 20, 40, 100, 150 and 200 mN) were applied to the receptive field and held for 10 sec each with a 1 min interval between successive stimuli.

For a separate experiment, additional C fibers were characterized and further subtyped by their heat and cold response properties. First, the receptive field was isolated from the bath solution using a plastic cylinder (2.4 mm inner diameter) and the receptive field was superfused with cold oxygenated buffer (ramp from 32°C to 2°C in ∼6 sec followed by a hold at 2°C for 2 min). Temperature was monitored in real time using a fast time constant thermocouple probe (Physitemp) adjacent to the skin. After a 2 min recovery period, warm (∼52°C) oxygenated buffer was superfused in the same way to apply a heat stimulus to the receptive field (ramp from 32°C to 50°C in ∼6 sec followed by a hold at 50° for 1 min). These fibers were subdivided into C-mechano (CM), C-Mechano Cold (CMC), C-Mechano Heat (CMH), or C-Mechano Heat-cold (CMHC) based on the modalities they responded to.

For another experiment, additional C fibers were characterized from inflamed mice. Their receptive fields were isolated with a steel cylinder and treated with HC-030031 (30 µM in 0.03% DMSO), A-425619 (1 µM in 0.03% DMSO) or vehicle (0.03% DMSO) diluted in warmed synthetic interstitial fluid. Fibers were treated with antagonist or vehicle for 2 min and then tested with increasing mechanical force (5–200 mN; 1 min interval) in the presence of compound. The compound was then removed and the receptive field washed. All C fibers within a given skin-nerve preparation were treated with HC-030031, A-425619 or vehicle, and the experimenter was blinded to chemical treatment.

### Chemicals

The TRPA1 antagonist HC-030031 was obtained from Hydra Biosciences. The TRPV1 antagonist A-425619 was obtained from Abbott Laboratories. All other chemicals were purchased from Sigma. Stock solutions of HC-030031 (100 mM in ethanol) and cinnamaldehyde (100 mM in ethanol) were made fresh daily.

### Data Analysis

For behavioral analyses, the average paw height, withdrawal threshold and paw withdrawal latency were calculated for each paw at baseline and 2 days following injection. Behavioral data at each time point were compared using a two-way repeated measures ANOVA followed by a Bonferroni post-hoc analysis.

Teased nerve fiber recordings were collected using a PowerLab4/sp, and were recorded and analyzed using the Chart program (v5.5.6; ADInstruments). The number of action potentials elicited by each stimulus was quantified. Comparisons across mechanical force or stimulus duration and between treatment groups were made using a two-way repeated measures ANOVA followed by a Bonferroni post-hoc analysis. CFA, saline, CFA+HC-030031, CFA+A-425619 and CFA+vehicle groups, though displayed separately in Figure 1 and Figure 3, were compared together for these analyses. Mechanical response distribution data (Figures 1D, 3D and 5F) represent a subset of the mechanical force response data (Figures 1A, 3A and 5A) and thus were not subjected to separate statistical analyses. Because von Frey filaments do not provide a continuous stimulus range for detecting mechanical threshold, these values were compared using a non-parametric Mann-Whitney U test. The overall incidence of spontaneous activity was compared using a Fisher’s exact test. The incidence of different subtypes of nerve fibers was compared using a Chi-square analysis.
